# Diversity of two-component systems: insights into the signal transduction mechanism by the *Staphylococcus aureus* two-component system GraSR

**DOI:** 10.12688/f1000research.5512.2

**Published:** 2014-11-17

**Authors:** Uzma Muzamal, Daniel Gomez, Fenika Kapadia, Dasantila Golemi-Kotra

**Affiliations:** 1 Departments of Biology, York University, Toronto, Toronto, ON, M3J 1P3, Canada; 2 Department of Chemistry, York University, Toronto, Toronto, ON, M3J 1P3, Canada

**Keywords:** Two-component systems, histidine kinases, response regulators, GraSR, Staphylococcus aureus, cationic antimicrobial peptides

## Abstract

The response to cationic antimicrobial peptides (CAMPs) in *Staphylococcus aureus* relies on a two-component system (TCS), GraSR, an auxiliary protein GraX and an ATP-binding cassette (ABC) transporter, VraF/G. To understand the signal transduction mechanism by GraSR, we investigated the kinase activity of the cytoplasmic domain of histidine kinase GraS and the interaction with its cognate response regulator GraR. We also investigated interactions among the auxiliary protein GraX, GraS/R and the ATPase protein of the ABC transporter, VraF. We found that GraS lacks autophosphorylation activity, unlike a similar histidine kinase, BceS, of *Bacillus subtilis*. In addition, the interaction between GraS and GraR is very weak in comparison to the stronger interaction observed between BceS and its conjugated response regulator, BceR, suggesting that CAMP signaling may not flow directly from GraS to GraR. We found that the auxiliary protein GraX interacts with VraF and GraR, and requires the histidine phosphotransfer and dimerization domain of GraS to interact with this protein. Further, VraF requires the GraS region that connects the membrane-bound domain with the cytoplasmic domain of this protein for interaction with GraS. The interactions of GraX with GraS/R and VraF indicate that GraX may serve as a scaffold to bring these proteins in close proximity to GraS, plausibly to facilitate activation of GraS to ultimately transduce the signal to GraR.

## Introduction

*Staphylococcus aureus*, a Gram-positive coccus, is both a commensal and a major human pathogen. As a commensal organism, it colonizes the skin and nares, and as a pathogen, it causes a variety of infections ranging from superficial skin abscesses to more serious diseases such as pneumonia, meningitis, endocarditis, septicemia, and toxic shock syndrome ^1^. The success of *S. aureus* as a pathogen relies on its ability to adapt to a wide variety of environmental conditions and to resist host innate immune defense mechanisms ^2^. The extensive use of antibiotics and the adaptability of *S. aureus* have led to the emergence of multidrug resistant strains in hospital and community settings ^3^.

In prokaryotes, environmental cues are channeled inside the cell via two-component systems (TCS). A typical TCS is composed of a membrane-bound histidine kinase (HK) sensor and a cognate response regulator (RR) protein. Each organism has a number of these systems that are specialized to respond to a specific cue, despite the conserved nature of domain organization and structural similarities among them ^4^.

The glycopeptide resistance- associated TCS GraSR, in which GraS is the histidine kinase and GraR is the response regulator protein, regulates the resistance to cationic antimicrobial peptides (CAMPs) in *S. aureus* ^5, 6^. *S. aureus* resistance to CAMPs involves an increase in the positive cell surface charge through D-alanylation of wall teichoic acid (WTA) and lysinylation of phosphatidyl-glycerol within the cell membrane ^7, 8^. Both processes are mediated by enzymes encoded by the *dltABCD* and *mprF* operons, respectively. GraR is directly involved in regulation of these two operons ^6, 9, 10^. Induction of these two operons is selective, and CAMPs such as RP-1 (platelets) and polymyxin B are capable of inducing *mprF* and *dltABCD*, but cationic molecules such as vancomycin, gentamicin or calcium-daptomycin are not ^11^.

*In vivo* studies showed that sensing and signaling of CAMPs in *S. aureus* relies on an ATP-binding cassette (ABC) transporter, encoded by the *vraFG* operon ^6^ and the third gene of *graRS* operon, *graX* ^12^. The *vraFG* operon is regulated directly by GraR, whereas the *graXRS* promoter is not regulated by GraR. The ABC transporter VraFG is composed of a membrane spanning domain protein, VraG, and an ATP-binding protein, VraF. It is proposed that the ABC transporter senses the presence of CAMPs and transduces the signal through GraSR with assistance from GraX ^12^. Resistance to CAMPs relies on GraR, but not on the ABC transporter, as overexpression of GraR reverses the effect of *vraFG*, *graS*, *graR* or *graX* deletion. Furthermore, the independence of CAMPs resistance from the ABC transporter suggests that VraG does not function as a detoxification element ^12^.

While the ABC transporter is considered to be the sensor for CAMPs, the mechanism of signaling through GraSR and the role of GraS in this process remains unknown. In a typical TCS, the HK becomes active upon sensing an extracellular stimulus in a process that requires phosphorylation of a conserved histidine residue in the cytoplasmic portion of the HK. The information is then transduced to the cognate RR in a second phosphorylation process, whereby a conserved aspartate residue of the receiver domain of the RR becomes phosphorylated. This phosphorylation step modulates the activity of RRs, which often have transcriptional regulatory activities ^4, 13^.

**Figure S1.**
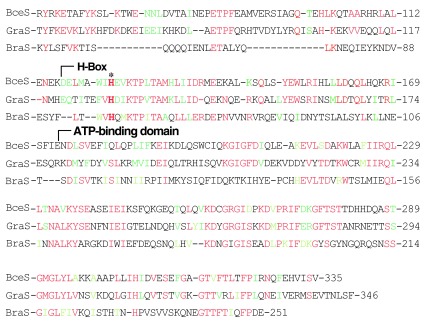
Sequence alignments among GraS, BceS and BraS.

Herein, we investigated the autophosphorylation activity of GraS and its interaction with GraR and took a close-up look at the interactions among GraS, GraR, GraX and VraF. For the latter, we cloned, expressed and purified for the first time the GraX and VraF proteins. As a reference in our study of signal transduction mechanism by GraSR, we used a homolog of GraSR in *Bacillus subtilis*, the BceSR TCS ^14^. There is a 36% sequence identity between GraS/BceS ( Figure S1) and 56% sequence identity between GraR/BceR. BceSR is involved in signaling and resistance to bacitracin. Like GraSR, BceSR relies on an ABC transporter for sensing bacitracin; however, unlike GraSR, its ABC transporter also acts as a detoxification element. Both RRs do not regulate the expression of their respective operons ^14, 15^.

Our study shows that the cytoplasmic domain of GraS, unlike BceS, does not have autokinase activity and does not interact with GraR. We show that the auxiliary protein GraX interacts with GraR and VraF. In addition, GraX and VraF interact with specific regions of GraS, and we propose that VraF may activate GraS. We see GraX as a bridge between GraS and GraR. Further, we show that there is no cross-talk between GraSR and BceSR, suggesting that, despite the similarities in primary sequences and domain organization between the respective HKs and RRs, other elements may determine the ultimate mechanism of signal transduction in a TCS.

## Materials and methods

***Chemical reagents and materials.*** Chemicals and antibiotics were purchased from Sigma (Oakville, Canada) or Thermo-Fisher (Whitby, Canada), unless otherwise stated. Chromatography media and columns were purchased from GE Healthcare (Quebec, Canada). Growth media were purchased from Fisher. *Escherichia coli* strains, NovaBlue and BL21(DE3), and cloning and expression plasmids were purchased from EMD4 Biosciences (New Jersey, USA). The pGEX-4T vector was purchased from GE Healthcare (Quebec, Canada). Restriction enzymes were obtained from New England Biolabs Canada (Pickering, Canada) or Thermo-Fisher. The [γ- ^32^P] ATP was purchased from Perkin Elmer LAS Canada Inc. (Toronto, Canada) or GE Healthcare. The Proteo Extract All-in-One Trypsin Digestion Kit was purchased from EMD4 Bioscience. The genomes of *S. aureus* strain Mu50 and *Bacillus subtilis* strain 168 were obtained from Cedarlane (Burlington, Canada). Oligonucleotides were acquired from Sigma (Canada).

### Cloning and expression of *bceR*, *graR*, *graR* ^N^ (1-134), and *graR* ^C^ (123-244), and purification of the respective gene products

The *bceR* gene was amplified from *B. subtilis* strain 168 genome, and *graR* was amplified from *S. aureus* strain Mu50 genome. The primer sets used for amplification of each gene are specified in Table 1. Cloning protocols for *bceR*, *graR* and *graR ^C^* were the same. Briefly, each amplicon was ligated to the blunt end sites of pSTBlue-1, and each construct was amplified in NovaBlue cells. The respective plasmids were digested with the appropriate set of restriction enzymes ( Table 1), and the inserts were ligated into pET26b(+) at the respective restriction sites. Cloning was confirmed by DNA sequencing (Core Facility, Biology, York University). The pET26b:: *bceR(or graR, graR ^C^)* construct was used to transform BL21(DE3). To clone *graR ^N^*, we introduced a stop codon after the 134 ^th^ residue using the Quick-Change mutagenesis kit (Agilent, Mississauga, Canada).

**Table 1.** Sequence of the primers used in this study.

Name	Primer Sequence (5′-3′)*	Restrict. enzyme
BceR Fwd	GC *CATATG*TTGTTTAAACTTTTGCTGATTGAAG	*NdeI*
BceR Rev	GC *GAATTC*TTAATCATAGAACTTGTCCTCTTCCTTC	*EcoRI*
GraR Fwd	GC *CATATG*ATGCAAATACTACTAGTAGAAGATGACAATAC	*NdeI*
GraR Rev	ACG *AAGCTT*TTATTATTCATGAGCCATATATCCTTTTCCTA	*HindIII*
GraR ^C^Fwd	GC *CATATG*GCTGAAGAAAAACGTACATTGACTTGG	*NdeI*
GraR ^C^Rev	CG *AAGCTT*TTATTCATGAGCCATATATCCTTTTCC	*HindIII*
BceS Fwd	G *GTCGAC*TCGCGTTTTATAAAAGCTTGAAAAC	*SalI*
BceS Rev	GC *GCGGCCGC*TCACACGCTTATGACATGTTC	*NotI*
GraS Fwd	ACG *GGATCC*GAAATAGAAGAAATTAAACATAAAGATTTAG	*BamHI*
GraS Rev	ACG *CTCGAG*TTTCATTTTGTAATGGGAAAATCAATC	*XhoI*
HGraS Fwd	CACCGAAATAGAAGAAATTAAACATAAAGATTTAG	
HGraS Rev	TTAAAATGACAAATTTGTCACTTCCG	
HGraX Fwd	AGCT *AAGCTT*ATGAAACCTAAAGTTTTATTAGCAG	*HindIII*
HGraX Rev	TCGA *CTCGAG*TCATTTAGTATATTTCATATTTTCTCC	*XhoI*
GraS ^CA^Fwd	*GGATCC*ATGTATTTTGATTACGTGTCACTT	*BamHI*
GraS ^CA^Rev	*CTCGAG*TTAAAATGACAAATTTGTCACTT	*XhoI*
GraS ^DHp-CA^FW	G *GGATCC*GTTGTTGAGCAACAGTTACAAT	*BamH1*
GraS ^DHp-CA^Rev	*CTCGAG*TTAAAATGACAAATTTGTCACTT	*XhoI*
GraX Fwd	ACG *CATATG*AAACCTAAAGTTTTATTAGCAGG	*NdeI*
GraX Rev	ACG *AAGCTT*TTATCATTTAGTATATTTCATATTTTCTCC	*HindIII*
VraF FW	CATG *CCATGG*TGGCAATTTTAGAAGTAAAAC	*NcoI*
VraF Rev	CG *GGATCC*TTAAAGGTCATAATTAACGCC	*BamHI*
VraFmFW	GGTGGCGTTAATTATGACCTTTTAGGATCCCCGAATTCGAGCTCC	
VraFmRev	GGAGCTCGAATTCGGGGATCCTAAAAGGTCATAATTAACGCCACC	

*Italicized sequences indicate the restriction sites. Abbreviations: Fwd, forward primer; RE, restriction enzyme; Rev, reverse primer

To express and isolate the target proteins, cell cultures were grown to exponential phase with an optical density at 600 nm (OD _600_) of ~0.6, induced with 0.5 mM isopropyl β-D-1-thiogalactopyranoside (IPTG, Rose Scientific, Edmonton, Canada), and shaken overnight at 18°C. Cells were harvested at 7,459 × *g*, resuspended in buffer I (20 mM Tris supplemented with 5 mM MgCl _2_, pH 7.0 for BceR and pH 7.5 for GraR), and sonicated to liberate the protein. Cellular debris was removed by centrifugation at 18,138 × *g* for 60 min. The supernatant was loaded onto a DEAE-Sepharose ^TM^ column equilibrated in buffer I, and the protein was eluted with a linear gradient of 500 mM Tris supplemented with 5 mM MgCl _2_ (pH 7.0 for BceR and pH 7.5 for GraR). In the case of BceR, fractions containing the protein were pooled and concentrated using Amicon ultracentrifugation membrane (ultracel 10K, Thermo-Fisher) and then loaded onto a heparin-sepharose affinity column equilibrated with buffer I. Protein was eluted with a linear gradient of 500 mM Tris supplemented with 5 mM MgCl _2_. For GraR, GraR ^N^ and GraR ^C^, as a second step of purification, we employed size-exclusion chromatography. Protein samples were loaded onto Sephacryl S-200 HiPrep 26–60 size-exclusion column (GE Healthcare) equilibrated with 50 mM Tris buffer (pH 7.4) and supplemented with 100 mM KCl and 5 mM MgCl _2_. The column was run at 1 mL/min. Fractions containing pure protein, as assessed by Coomassie-stained SDS-PAGE, were collected together and concentrated.

Protein concentrations were determined by the Bradford reagent (GE Healthcare). The molecular masses of the isolated proteins were confirmed by electron-spray ionization mass spectrometry (ESI-MS) at the Advanced Protein Technology Centre, Hospital for Sick Kids (Toronto, Canada).

### Cloning and expression of glutathione S-transferase ( *gst*)-tagged *bceS*, - *graS*, - *graS* ^CA^, - *graS* ^DHp-CA^, and purification of the respective gene products

The nucleic acid sequence encoding the cytoplasmic region of *bceS*, spanning residues 62 to 334, ** was amplified from *B. subtilis* 168 genome. The nucleic acid sequence encoding the cytoplasmic domain of *graS*, spanning residues 77–346, was amplified from *S. aureus* Mu50 genome. The same genome was used to amplify the nucleic acid sequence encoding the ATP-binding domain of GraS (GraS ^CA^), spanning residues 181–346. We also amplified the nucleic acid sequence encoding the dimerization and histidine phosphorylation (DHp) domain and the CA domain of *graS*, spanning residues 110–346. This construct is referred to as GraS ^DHp-CA^. The primer sets used for amplification of the above *graS* regions are specified in Table 1. We used the pGEX-4T-1 vector to clone the N-terminal GST-fusion proteins of GraS, GraS ^CA^, GraS ^DHp-CA^ and BceS. We also cloned the GraS cytoplasmic domain with a hexa-histidine tag on its NH _2_-terminus, using the pET151/D-TOPO vector.

Each gene was ligated to the blunt-end sites of pSTBlue-1, and this construct was amplified in *E. coli* NovaBlue. Each plasmid was isolated using the GeneJet ^TM^ plasmid extraction kit and digested with the appropriate set of restriction enzymes to liberate the respective insert, which was then ligated into pGEX-4T-1 at the respective restriction sites ( Table 1). The pGEX-4T-1:: *bceS*( *graS, graS* ^CA^ or *graS* ^DHp-CA^) plasmid was introduced into *E. coli* BL21(DE3). Expression and purification of GST-GraS, GST-GraS ^CA^, GraS ^DHp-CA^ or GST-BceS was carried out in the same way. Briefly, protein expression was initiated with 0.5 mM IPTG once the cell cultures reached OD _600_ ~0.6. Induction proceeded overnight at 18°C. Cells were spun down at 7,459 × *g* for 20 min, resuspended in 1 × phosphate-buffered saline buffer (PBS, pH 7.4), and then sonicated to liberate the cell contents. Cellular debris was removed by centrifugation at 18,138 × *g* for 60 min. Purification of each protein was carried out using glutathione-sepharose affinity resin (GE Healthcare). The target protein was eluted with 10 mM reduced glutathione in 50 mM Tris (pH 8.0). Fractions containing the protein were collected together.

Expression of His-GraS was performed in the same manner as for GST-GraS. The cell pellet was resuspended in 20 mM sodium phosphate pH 8.0 buffer supplemented with 300 mM NaCl and 20 mM imidazole. The cells were processed as described above. The supernatant was loaded onto a Ni-NTA (nickel-nitrilotriacetic acid) column (Qiagen), and protein was purified using a linear gradient of imidazole.

### Cloning and expression of *graX* and purification of the respective gene product

The full length *graX* gene was amplified from *S. aureus* Mu50 genome using the primers provided in Table 1. The *graX* gene was cloned into pET26b between *NdeI* and *HindIII* restriction sites. The *graX* gene was also cloned to the C-terminus of MAT-Tag (HNHRHKH) and -FLAG (DYKDDDDK) epitopes using the pT7 MAT-tagFLAG-1 vector (Sigma). In this case, *graX* was inserted between the *HindIII* and *XhoI* restriction sites of the vector ( Table 1). Each expression vector, pET26b:: *graX* or pT7MAT-tagFLAG-1:: *graX*, was introduced into *E. coli* BL21(DE3).

GraX and MAT-FLAG-GraX were expressed in the same way. Cell cultures were grown to an OD _600_ ~0.6 at 37°C. At this point, the cultures were cooled at 4°C, induced with 0.5 mM IPTG, and allowed to express protein over 16 hours at 18°C. Cells were harvested by centrifugation at 7,459 × *g* for 20 min and resuspended in 50 mM sodium phosphate pH 7.2. For MAT-FLAG-GraX, the buffer was supplemented with 300 mM NaCl, and the pH was adjusted to 8.0. Cellular content was liberated through sonication, and the resulting cell lysate was centrifuged at 18,138 × *g* for 1 hour to remove the cellular debris. For GraX, the supernatant was loaded onto SP-Sepharose cation exchange column. GraX protein was eluted using a linear gradient of 0 to 1 M sodium chloride in 50 mM sodium phosphate pH 7.2. The fractions containing GraX were pooled and concentrated using Amicon stirred cell concentrator (EMD Millipore). The protein sample was dialyzed against 50 mM Tris, pH 7.4, 5 mM MgCl _2_ and 300 mM NaCl. MAT-FLAG-GraX was purified using a Ni-NTA column (Qiagen). The protein was eluted with a linear gradient of imidazole from 10 mM to 300 mM imidazole over 5 column volume.

### Cloning and expression of six-histidine-tagged *vraF*, and purification of the respective gene product

The full length *vraF* DNA sequence (762 bp) was amplified from *S. aureus* Mu50 genome. The primers designed to clone *vraF* into the pET24d expression vector harbor the restriction sites of *NcoI* and *BamHI* ( Table 1). The amplicon was digested with *NcoI* and *BamHI* and ligated into the pre-treated pET24d vector with the same restriction enzymes. The resulting construct, pET24d:: *vraF* was introduced into *E. coli* Nova Blue by heat shock at 42°C. The *vraF* insertion into the pET vector and the correctness of the gene sequence was confirmed by DNA sequencing (The Centre for Applied Genomics, The Hospital for Sick Kids, Toronto, Canada). The pET24d:: *vraF* construct was introduced into the expression host *E. coli* BL21(DE3) by heat shock.

To enable cloning of VraF fused to a 6 × histidine tag (His _6_-tag) on its C-terminus (His-VraF), we removed the stop codon on *vraF* to enable translation of a linker region and the 6 histidine tag in pET24d, downstream of *vraF*. The mutagenesis primers are provided in Table 1. The QuickChange ^®^ mutagenesis kit was used to carry out the mutation (Agilent Technology). The mutation was confirmed by DNA sequencing.

Untagged *vraF* expression was attempted with 0.5 mM or 1 mM IPTG concentration at 18°C or 25°C for 18 hrs, in the absence or presence of 0.5 M sorbitol and 3 mM betaine. However, all these conditions resulted in expression of the protein trapped in inclusion bodies.

Over-expression of the C-terminal His _6_-tagged *vraF* was carried out with 0.1 mM IPTG, over 16 hrs by shaking at 16°C. Briefly, 1 mL of overnight grown cell culture of *E. coli* BL21(DE3) was used to inoculate 1 L of Terrific Broth medium, supplemented with 2.5 M sorbitol and 3 mM betaine. When cell culture reached an OD _600_ ~0.6, the cell culture was cooled down at 4°C and IPTG was added to a final concentration of 0.1 mM to induce protein expression. The cells were induced over 16 hours at 16°C and subjected to continuous shaking at 200 rpm. They were then collected by centrifugation at 7,459 × *g* for 20 min. The cell pellet was resuspended in Buffer A (50 mM sodium phosphate, 100 mM NaCl, 10 mM imidazole, pH 7.0 buffer). The cells were lysed by sonication and cell debris was removed by centrifugation at 18,138 × *g* at 4°C for 1 hr.

Purification of His-VraF was carried out using the batch method. The cell lysate was incubated with 1 mL Ni-NTA resin (Qiagen) for 1 hr at 4°C. The cell lysate-resin mixture was loaded into a column. The resin was washed with Buffer A until no protein was washed out from the column. Elution of VraF was carried out using a step gradient of imidazole in Buffer A: 4 × 1.5 ml 100 mM imidazole, 4 × 1.5 ml 150 mM imidazole; 4 × 1.5 ml 200 mM imidazole and 4 × 1.5 ml 300 mM imidazole.

### Autophosphorylation of histidine kinases

Autophosphorylation of GST-GraS or GST-BceS was performed as described previously with minor modifications ^16^. Purified GST-BceS, GST-GraS or His-GraS (5 µM) was equilibrated in the phosphorylation buffer (PB: 50 mM Tris, 50 mM KCl, 5 mM MgCl _2_, pH 7.4) at a final volume of 10 µL. The PB was supplemented with 20 mM CaCl _2_ (BceS) or 10 mM CaCl _2_ (GraS). The reaction was initiated by adding [γ- ^32^P] ATP (10 Ci/mmol or 3000 Ci/mmol) at room temperature. Aliquots were removed at different time intervals, and the reactions were quenched by the addition of 5 × SDS sample buffer (2.5% SDS, 25% glycerol, 125 mM Tris-HCL, pH 6.8, 0.0025% bromophenol blue). Samples were analyzed by 12.5% SDS-PAGE. The gels were dried and exposed to an autoradiography cassette, which was scanned using TYPHOON Trio ^+^ (GE Healthcare). The band intensities were analyzed by NIH ImageJ software (version 1.45s). The band intensities were plotted against time, and these curves were referred to as progress curves. The rate constant was determined by plotting the intensity values against a first-order integrated rate law with the equation *I* = A * (1 – *e ^–kt^*) where, *I* is the intensity of the band, *k* is the rate constant, *t* is time, and *A* is the proportionality constant between the intensity and concentration of GST-BceS-P. Erithacus GraFit software (version 5.0.10) was used to fit the experimental data. The phosphotransfer between the HK and its cognate RR, and phosphorylation of RRs by small molecule phosphate donors such as acetyl phosphate were carried out as described previously ^16^.

### Circular Dichroism (CD) experiments

To investigate whether the purified proteins were folded properly, we collected the CD spectrum (200–240 nm) of each protein on a Jasco J-810 instrument (Jasco, Tokyo, Japan) at 22°C using a cuvette with a 0.1cm path length. The CD spectra were collected in 30 mM Tris (pH 7.0) supplemented with 5 mM MgCl _2_. The final spectra were corrected for buffer contribution.

### Investigation of protein-protein interactions by pull-down assays

Glutathione-sepharose affinity resin (75 µL) was equilibrated in 1 × PBS buffer. GST-BceS, GST-GraS, GST-GraS ^CA^ or GST-GraS ^DHp-CA^ was incubated with the resin for 30 min at room temperature. The flow-through was collected, and the resin was washed with 1 × PBS buffer until no protein eluted. At this point, GST-BceS-bound resin was incubated with BceR at a 1:1.6 ratio, the GST-GraS-bound resin was incubated with GraR at a 1:2 or 1:5 ratio, GraX at a 1:1.3 ratio, or His-VraF at 1:1.3 ratio, the GST-GraS ^CA^-bound resin was incubated with His-GraX at 1:1.6 ratio, or His-VraF at 1:2 ratio, and GST-GraS ^DHp-CA^ was incubated with His-VraF at 1:2 ratio. The incubation time in all the cases was 30 min at room temperature. Subsequently, the resins were washed five to seven times with 200 µL of 1 × PBS buffer. The proteins were eluted with 200-µL aliquots of 10 mM reduced glutathione in 50 mM Tris (pH 8.0). Flow-through fractions, wash fractions, and elution fractions were analyzed by 12.5% SDS-PAGE. The immobilized GST-BceS or GST-GraS were also incubated with bovine serum albumin (BSA) to investigate potential non-specific interactions with BceR and GraR, respectively. In addition, the resin itself was incubated with the prey proteins BceR, GraR or GraX to investigate for non-specific interactions of these proteins with resin.

A similar protocol was used to investigate the interaction between GraX and GraR, and His-VraF and GraX. In the former case, MAT-FLAG-GraX was immobilized onto Ni-NTA agarose resin, GraR was added at a 1.2:1 ratio. In the latter case, His-VraF was immobilized onto Ni-NTA agarose resin and untagged GraX was added at 1:2 ratio. The elution fractions were analyzed by 15% SDS-PAGE.

### Oligomerization state of proteins

The oligomerization states of GraR, GraR ^N^ and GraR ^C^ were determined by the high-performance liquid chromatography (HPLC) size-exclusion column TSK Gel (7.8mm × 30cm, 5µm). The column was calibrated with the standard proteins: aprotonin (6.5 kD), carbonic anhydrase (29 kD), ribonuclease A (37 kD), ovalbumin (45 kD), and conalbumin (75 kD). The molecular masses of the target proteins were determined from the standard curve (log of molecular mass versus retention time). The oligomerization state of GraX was investigated by SDS-PAGE in the presence and absence or dithiothreitol (DTT). The low solubility level of GraX prevented investigation of oligomerization by size exclusion chromatography.

### DNase I footprinting experiments

The promoter region of *vraFG* ( *P*_*vraFG*_), spanning between +28 to -168 with respect to the transcription start site ^15^, was amplified and used to probe the DNA-binding activity of GraR. The *graSR* promoter region spanning -115 to +75 was amplified using the primers Dir-5′-CG *GAATTC*ATTGAAATGAAATTTTCTACA TC-3′ and Rev-5′-CG *GGATCC*TTTAGGTTTCATCTAAAATACTCC-3′. Prior to amplification, the primers were 5′ end-labeled with [γ- ^32^P]ATP (3000 Ci/mmol) using T4 polynucleotide kinase. The DNase I footprinting was carried out as described previously ^17^. The gels were dried and exposed to an autoradiography cassette, which was scanned using TYPHOON Trio ^+^ (GE Healthcare). The footprinting gels were analyzed by NIH ImageJ software (version 1.45s). The DNase I footprinting data were used to assess the dissociation constant as the GraR concentration that provided 50% protection.

## Results

### Isolation of target proteins

GraS (346 amino acids) and BceS (334 amino acids) are similar HKs; they share 36% sequence identity and both use an ABC transporter for signaling ^14, 15^. They consist of a membrane bound domain (BceS: spanning residues 1–55; GraS: spanning residues 1–63) and a cytoplasmic domain referred to as the kinase domain (BceS: spanning residues 105–336; GraS: spanning residues 110–346). We used the amino acid sequence alignments of both proteins to determine the N-termini of each protein construct so that similar regions of these proteins were cloned. Cloning of similar regions of the cytoplasmic portions of GraS and BceS will allow a direct comparison of their functions. The cytoplasmic domains of GraS (77–346) and BceS (62–334) were independently fused at the COOH-terminus of GST, and the proteins were purified to homogeneity. Two other constructs of GraS were fused to the COOH-terminus of GST: GraS ^DHp-CA^, spans residues 110 to 346 and lacks the membrane domain and the linker region; and GraS ^CA^, spans residues 181 to 346 and harbors only the ATP-binding domain of GraS. In addition, the cytoplasmic domain of GraS was also fused to a hexa-histidine tag at its NH _2_-terminus.

**Figure 1.**
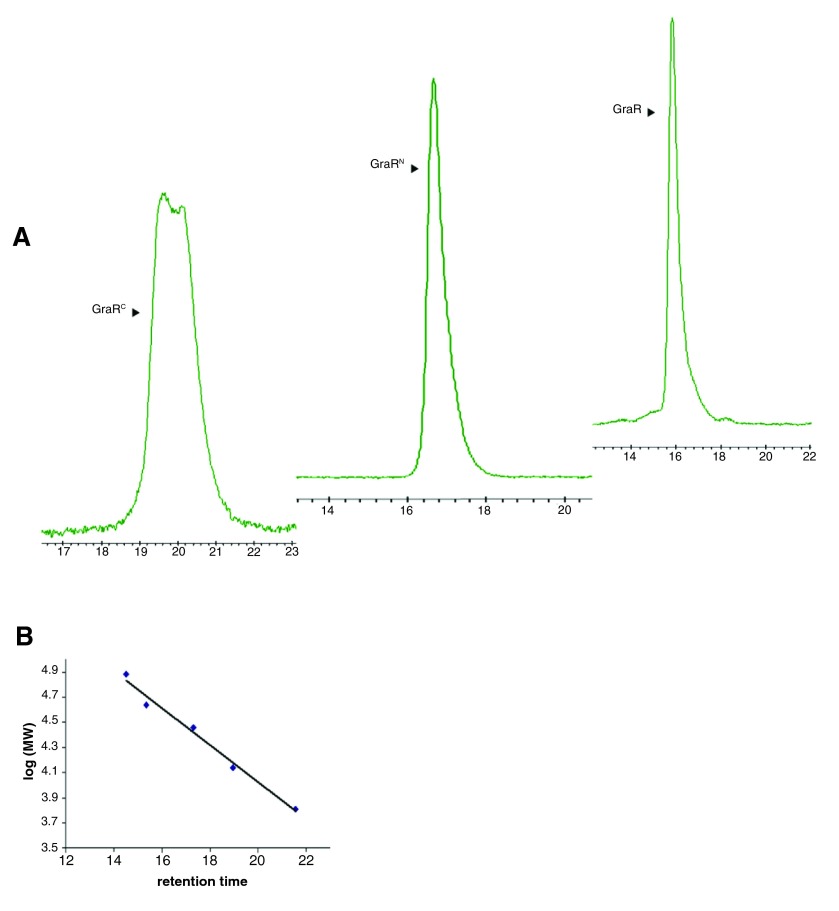
Oligomerization states of GraR, GraR ^C^ and GraR ^N^ proteins as assessed by size-exclusion chromatography. **A**) The chromatographs represent the elution profile of the GraR, GraR ^N^ and GraR ^C^ proteins on a HPLC size-exclusion TSK Gel (7.8mm × 30cm, 5µm) column calibrated using the following proteins: aprotonin (6.5 kDa), carbonic anhydrase (29 kDa), ribonuclease A (37 kDa), ovalbumin (45 kDa) and conalbumin (75 kDa). **B**) Calibration graph of log of molecular masses against the retention time of each protein standard.

Cloning of *bceR*, *graR*, *graR* ^N^, and *graR* ^C^ encoded, respectively, proteins with no extra amino acids on their NH _2_- or COOH-termini. The proteins were purified in two steps. GraR, GraR ^N^, and GraR ^C^ were purified to homogeneity, and BceR was purified up to 90% purity. The identities of the proteins were confirmed by trypsin digestion and liquid chromatography mass spectrometry (LC/MS), and their molecular masses were confirmed by electrospray-ionization MS at Toronto’s Sick Kids Advance Protein Technology Center (Toronto, Canada). GraR, GraR ^N^, and GraR ^C^ are monomers in solution, as indicated by the size exclusion chromatography ( Figure 1).

Cloning of *graX* was performed into two ways to result in production of a tagless GraX or fused to the COOH-terminus of a MAT-FLAG tag. In both cases, the proteins were purified to 90%. SDS-PAGE indicated presence of GraX dimers in solution (data not shown). Dimeric GraX species were removed in the presence of DTT, suggesting that the single cysteine residue of GraX could mediate dimerization through disulfide bond formation. Aggregation of GraX at high protein concentrations (>50 µM) hampered our efforts to determine the oligomerization state of GraX by size exclusion chromatography.

Production of tagless VraF was in good amount but in insoluble form. We fused a (His) _6_-tag to the COOH-terminus of VraF which increased the solubility of the produced protein and resulted in its purification at 90% homogeneity.

### GraS lacks autophosphorylation activity in contrast to BceS

Cytoplasmic domains of histidine kinases have been used as models to study the autokinase activities of full-length proteins ^18, 19^. Herein, we undertook the study of the autokinase activities of BceS and GraS. Efforts to express full-length *graS* resulted in production of GraS in insoluble form.

**Figure 2.**
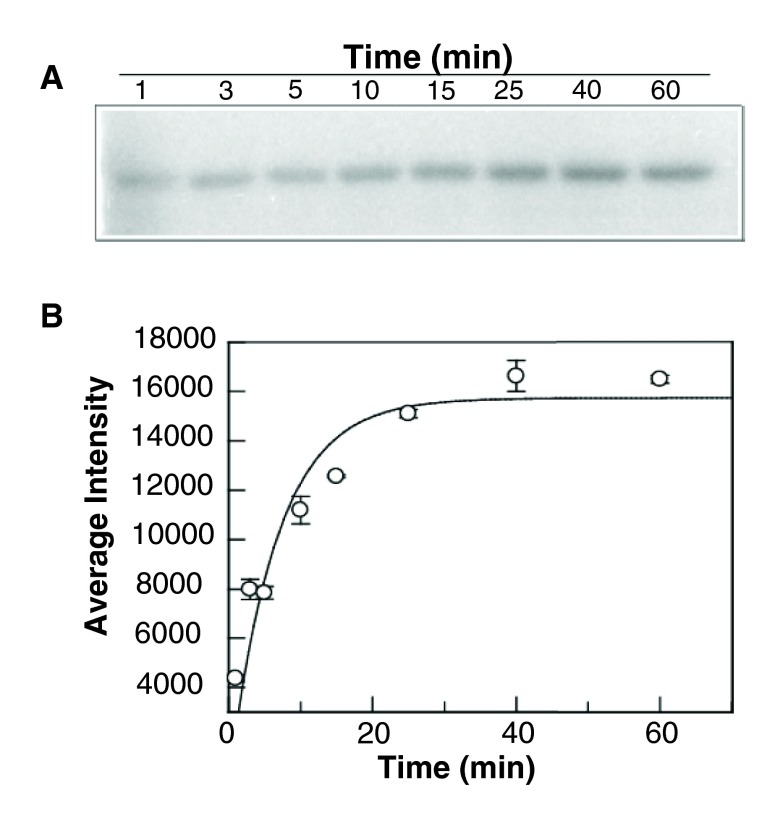
Autophosphorylation of BceS. ( **A**) GST-BceS (5 µM) was incubated with [γ- ^32^P] ATP (1 mM) in 50 mM Tris, 50 mM KCl, 20 mM CaCl _2_, and 5 mM MgCl _2_ (pH 7.4). Reactions were quenched at different incubation times and analyzed by 12.5% SDS-PAGE. ( **B**) The experimental data obtained in ( **A**) were quantified using ImageJ and plotted against the incubation time (the error bars represent the standard deviations calculated from three independent experiments). The data were fitted to the equation given in the Experimental Section.

**Figure 3.**
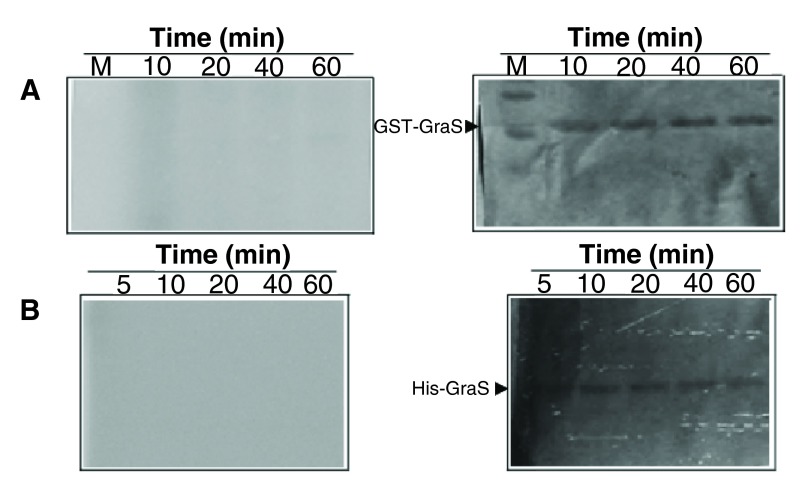
Attempted autophosphorylation of GraS. ( **A**) GST-GraS (5 µM) in 50 mM Tris, 50 mM KCl, 10 mM CaCl _2_, and 5 mM MgCl _2_ (pH 7.4) was incubated with 10 µM γ- ^32^P-ATP. The reaction was quenched at different time intervals. ( **B**) Autophosphorylation of His-GraS under the same conditions as in ( **A**). Left panel represent phosphor imaging and right panels represent the Coomassie staining of the SDS-PAGE gels.

**Figure 4.**
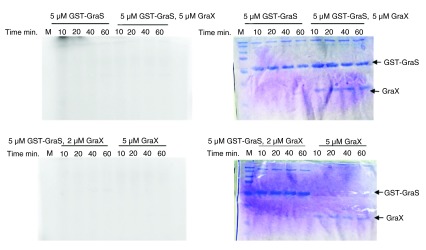
Attempt to phosphorylate GST-GraS in the presence of GraX. GST-GraS 5 µM alone or along with 2 or 5 µM GraX was incubated at room temperature for 30 min before adding [γ- ^32^P] ATP. GraX (5 µM) was used as control. Reactions were quenched at different time intervals and analyzed by 12.5% SDS-PAGE. On left side are shown the radiogram images of the SDS-PAGE and on the right the Coomassie stained images of the SDS-PAGE.

Autophosphorylation of GST-BceS in the presence of 1 mM ATP, at room temperature, showed a sharp increase in the signal intensity during the first 15 min, followed by saturation over the next 45 min ( Figure 2). The pseudo-first order rate constant was determined to be 0.15 ± 0.03 min ^-1^. In contrast, GraS did not undergo phosphorylation either as GST-GraS or as His-GraS ( Figure 3A, B). We tried different ATP concentrations and different concentrations of GST-GraS or His-GraS, but no autophosphorylation activity of GraS was observed (data not shown). As a positive control in our experiments, we used GST-VraS ^16^. In addition, we investigated the effect of the auxiliary protein GraX on the autokinase activity of GST-GraS and did not observe any effect ( Figure 4).

**Figure 5.**
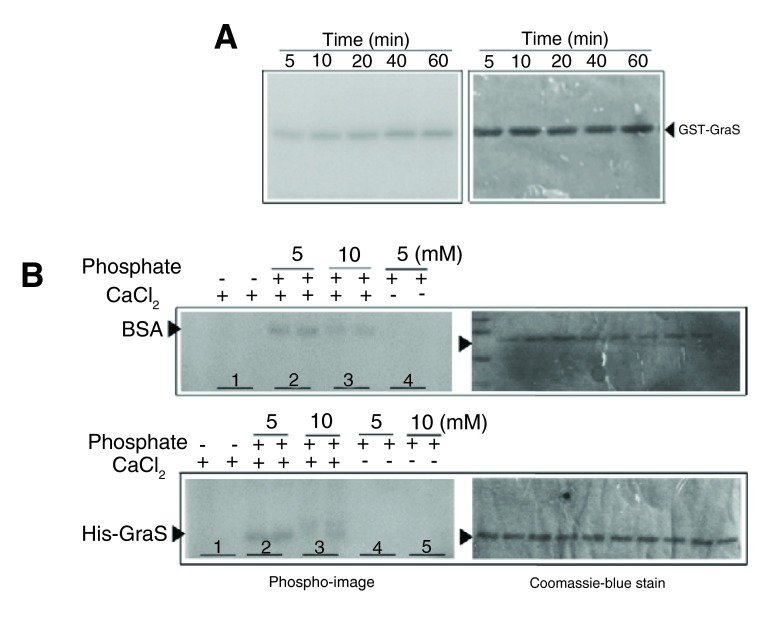
Investigation of CaCl _2_ and sodium phosphate roles on non-specific phosphorylation of GraS by ATP. ( **A**) GST-GraS (5 µM) in the phosphorylation buffer supplemented with 10 mM CaCl _2_ and 10 mM sodium phosphate incubated with [γ-P ^32^] ATP. The samples were resolved in a 12.5% SDS-PAGE. Gels were analyzed by autoradiography. ( **B**) His-GraS (5 µM) or BSA (5 µM) was incubated in the phosphorylation buffer supplemented with different concentrations of sodium phosphate in the presence or absence of 10 mM CaCl _2_ and incubated with [γ-P ^32^] ATP. Samples at a particular condition were quenched at 30 and 60 min (respectively, first and second lane of each sample) and analyzed by 12.5% SDS-PAGE. Gels were analyzed by autoradiography.

Interestingly, we noted that presence of sodium phosphate and CaCl _2_ in the phosphorylation buffer resulted in false-positive phosphorylation of His-GraS or GST-GraS, and a similar observation was made for BSA ( Figure 5). This could be due to the formation of insoluble calcium phosphate species in the buffer. Proper buffer exchange of the GST-GraS into Tris-HCl buffer eliminated the non-specific phosphorylation of GraS ( Figure 5).

### BceR undergoes phosphorylation by its cognate kinase, BceS

**Figure 6.**
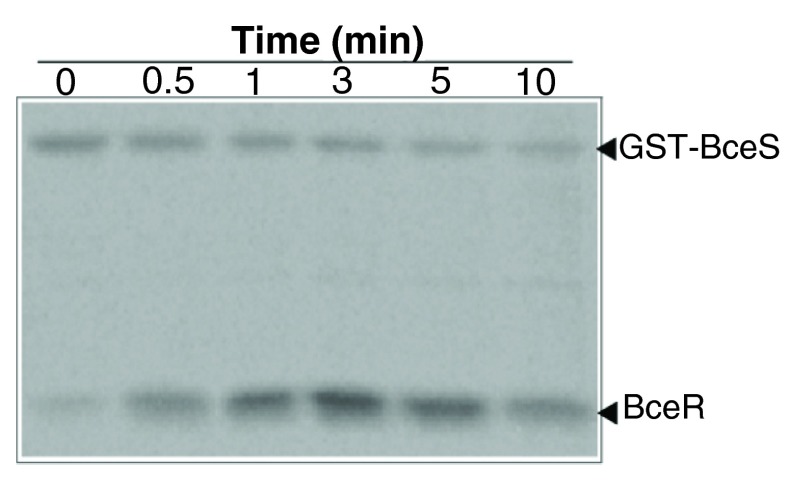
Phosphotransfer between BceS and BceR. BceR (5 µM) was incubated with GST-BceS- ^32^P (1 µM) at different time intervals in 50 mM Tris, 50 mM KCl, 20 mM CaCl _2_, and 5 mM MgCl _2_ (pH 7.4). The samples were analyzed by 15% SDS-PAGE. Gels were analyzed by autoradiography.

The autophosphorylation of BceS allowed us to investigate the phosphotransfer between the kinase and its cognate RR, BceR. Incubation of the phosphorylated GST-BceS (2 µM) with BceR (10 µM) resulted in the phosphotransfer of the phosphoryl group to BceR ( Figure 6). The maximum amount of phosphorylated BceR was achieved within 5 min, at which point phosphorylated-BceR species started to decrease, suggesting that GST-BceS has phosphatase activity in addition to its kinase activity in analogy with the observations made for VraSR system ^16^. Because of the high sequence homology between GraR and BceR and their similar functions, we investigated whether there was cross-talk between BceS and GraR. Incubation of phosphorylated GST-BceS with GraR did not result in the phosphorylation of GraR (data not shown).

**Figure 7.**
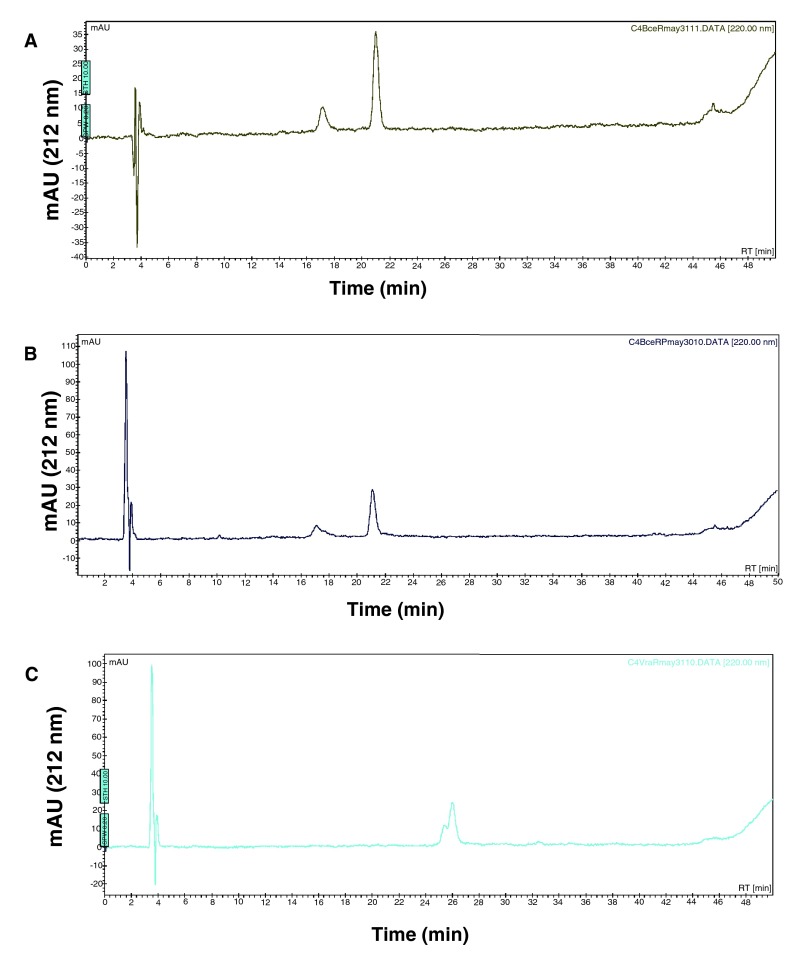
Attempts to phosphorylate BceR with acetyl phosphate. A 50 µL reaction was prepared containing 9.52 µM of BceR incubated in the absence ( **A**) or presence of 50 mM of acetylphosphate in 50 mM Tris, 50 mM KCl, and 20 mM MgCl _2_ ( **B**) (The smaller peak that elutes at 17 min is an impurity in both graphs **A** and **B**). The reaction mixtures were incubated at 37°C for 1 hour and then 40 µL were loaded onto an HPLC C4 reverse phase column. Elution of the protein was monitored. As a control, we monitored phosphorylation of VraR under the same conditions ( **C**) (The smaller peak that elutes at 25.2 min corresponds to the phosphorylated VraR).

**Figure 8.**
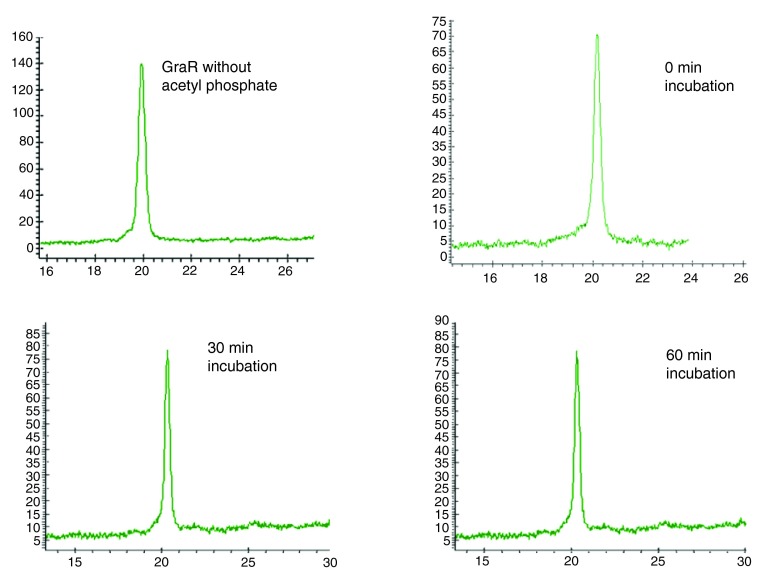
Attempts to phosphorylate GraR with acetyl phosphate. Full length GraR (40 µM) was incubated with 50 mM acetyl phosphate in 50 mM Tris, 50 mM KCl, and 20 mM MgCl _2_ for different time intervals at 37°C. The samples were analyzed on an HPLC C4 reverse phase column by monitoring the absorbance at 212 nm (Y-axis; X-axis is the elution time in min).

**BceR and GraR do not undergo phosphorylation by acetyl phosphate.** BceR and GraR were incubated with acetyl phosphate under conditions known to phosphorylate VraR ^16^. Samples were analyzed in a C4 reverse-phase column connected to a HPLC (Prostar, Agilent). We found that BceR and GraR did not undergo phosphorylation. Under the same conditions, VraR underwent phosphorylation ( Figure 7, Figure 8), as reported before ^16^.

### BceR interacts strongly with its cognate kinase, unlike GraR

**Figure 9.**
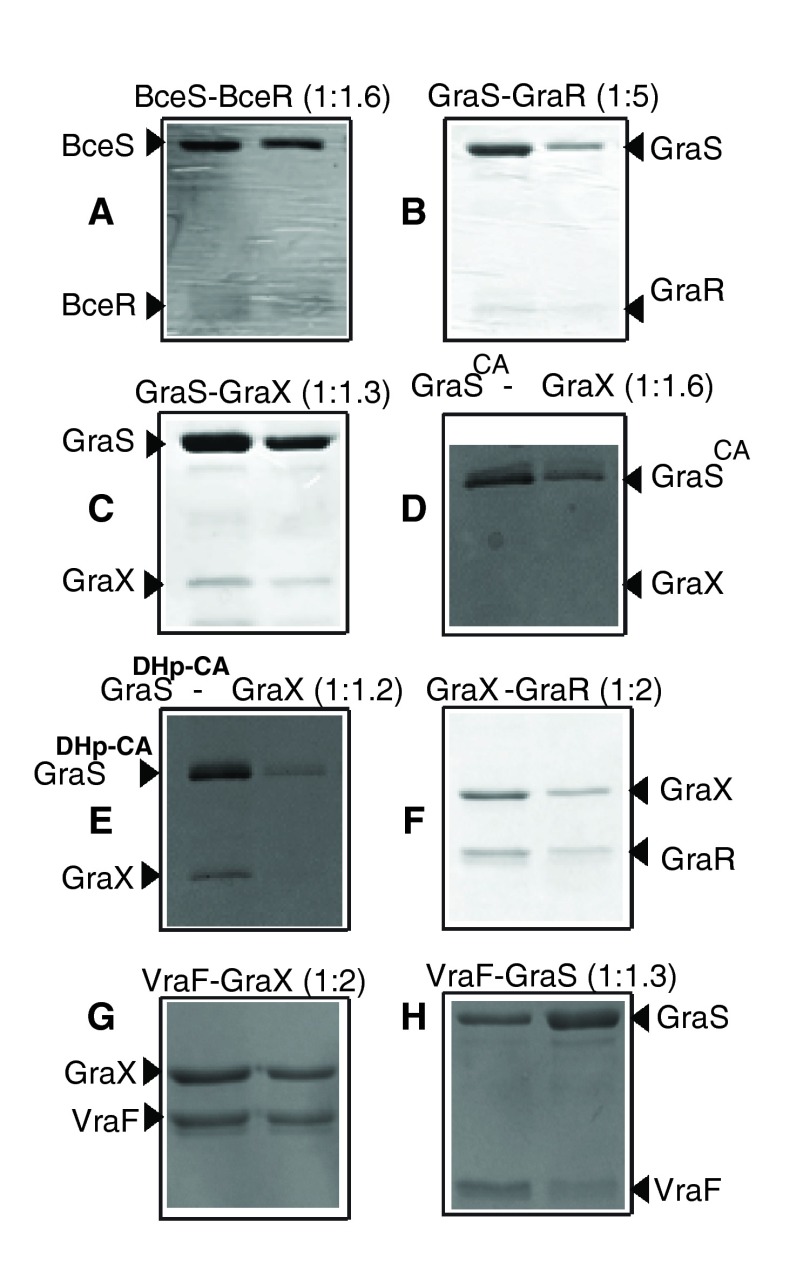
Pull-down assays. The bait proteins: GST-BceS ( **A**), GST-GraS ( **B**), GST-GraS ( **C**), GST-GraS ^CA^ ( **D**), MAT-FLAG-GraX ( **E**, **F**), His-VraF ( **G**, **H**), were immobilized onto their respective resins, glutathione resin ( **A**– **D**) or Ni-NTA resin ( **E**– **H**). The resins were washed to remove unbound proteins. The prey proteins, BceR ( **A**), GraR ( **B**, **F**), GraX ( **C**, **D**, **G**) or GraS ( **H**) were incubated with the resins at room temperature, and the unbound proteins were removed through seven successive washes. The protein contents of two successive elution fractions were analyzed by 12.5% SDS-PAGE. The ratios of bait protein to prey protein are given in parenthesis in each case.

**Figure 10.**
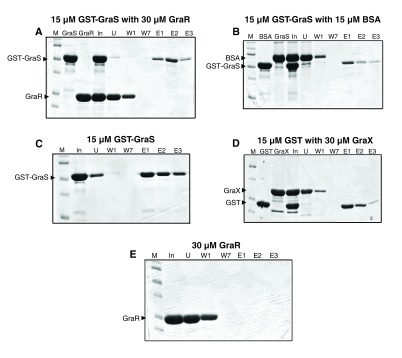
Investigation of interactions amongst GraS, GraX, GraR and BSA. ( **A**) GST-GraS was immobilized onto the glutathione resin and incubated with GraR. The resin was washed seven times, and finally eluted three times with 10 mM glutathione. Lanes: M, marker; GraS, protein prior to immobilization; GraR, protein prior to incubation with resin; In, incubation mixture in the absence of resin; U, unbound fraction; W1 and W7, fractions collected through washes of the resin; E1, E2, E3, three subsequent elution fractions collected when resin was incubated with elution buffer. ( **B**) GST-GraS was immobilized onto the glutathione resin and incubated with BSA. Similar protocol of washing and elution was used as in ( **A**). ( **C**) GST-GraS was immobilized onto the glutathione resin followed by the protocol given in ( **A**). ( **D**) GST was immobilized onto the glutathione resin, followed by the protocol given in ( **A**). ( **E**) GraR was immobilized onto the Ni-NTA resin, washing and elutions were done as in ( **A**).

To investigate the interactions between the histidine kinases and their cognate response regulators, GST-GraS or GST-BceS was immobilized onto the glutathione resin. The resin-bound GST-GraS or GST-BceS was incubated with GraR or BceR, respectively. BceR co-eluted with BceS during the elution steps ( Figure 9A). By contrast, GraR eluted during the washing steps when incubated with GraS at 1:2 ratio ( Figure 10A). In the case when GraR concentration was 5-fold more than GraS, we observed co-elution of GraR with GST-GraS during the elution steps ( Figure 9B).

To ensure the specificity of the interactions in our experimental set-up, we used BSA as the prey. In this case, BSA (at a ratio of 1:1) was not retained by the resin-bound GST-GraS. Elution with 10 mM reduced glutathione released only GST-GraS ( Figure 10B).

### GraX interacts with GraS and GraR

To investigate the interaction between GraS and GraX, pull-down experiments were carried out by incubating resin-bound GST-GraS with GraX at the 1:1.3 ratio. No GraX eluted from the column during the seven washes of the resin. GST-GraS and GraX co-eluted in the first fraction of the elution step ( Figure 9C). We repeated the experiments by immobilizing GST only and incubating the resin-bound GST with GraX. Using the same protocol for washing and elution, we observed that GraX eluted in the first three washes, and GST eluted alone when the resin was incubated with 10 mM glutathione buffer ( Figure 10).

The full-length GraS protein consists of two domains, the N-terminal dimerization and histidine phosphotransfer domain (DHp) and the C-terminal ATP-binding domain (CA) ^20^. The DHp domain harbors the conserved histidine residue, His129, that undergoes phosphorylation upon activation of the kinase, and the CA domain binds to ATP and catalyzes the transfer of the γ-phosphoryl group of ATP to the conserved histidine residue. To determine which GraS domain interacts with GraX, we investigated its interaction with GraS ^CA^ (181–346) and GraS ^DHp-CA^ (110–346) using the pull-down assay. In this experiment, GST-GraS ^CA^ was immobilized on the resin and incubated with GraX. At a ratio of 1:1.2 GraX:GST-GraS ^CA^, we did not observe interaction between GraX and GST-GraS ^CA^ ( Figure 9D). However, at the same ratio, GraX was pulled down by GraS ^DHp-CA^ ( Figure 9E). This is a strong indication that GraX requires the DHp domain of GraS for interaction.

To investigate the interaction between GraX and GraR, MAT-FLAG-GraX was immobilized onto Ni-NTA resin. Immobilized GraX was incubated with GraR at a 1:1.2 ratio. GraR co-eluted with GraX during the elution steps ( Figure 9F). GraR was not retained by the resin alone ( Figure 10E).

### VraF interacts with GraX and GraS

His-VraF immobilized on Ni-NTA column was able to recruit GraX when incubated at 1:2 ratio with this protein ( Figure 9G). To investigate the interaction of VraF with GraS, we looked at three different constructs of GraS: the full-length cytoplasmic domain of GraS which harbors partially the linker (residues 77–110) that connects the cytoplasmic domain to the membrane binding domain; GraS ^DHp-CA^ which lacks completely the linker; and GraS ^CA^ that harbors the ATP-binding domain. The pull down experiments showed that only full-length cytoplasmic domain of GraS was pull-downed by VraF ( Figure 9H), indicating that VraF requires the linker region of GraS (77–110) for interaction.

### CD spectra of the target proteins

**Figure 11.**
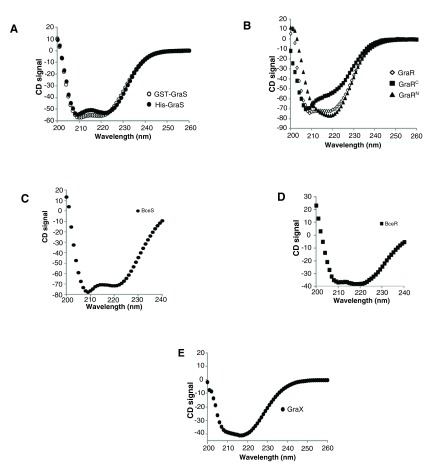
CD spectra of target proteins. CD spectra of GST-GraS (20 μM), His-GraS (20 μM), GraR (30 μM), GraRC (40 μM), GraRN (40 μM), BceR (20 µM), BceS (40 µM) and GraX (18 μM) were obtained in 30 mM Tris (pH 7.0) supplemented with 5 mM MgCl _2_. The spectra were corrected for the buffer contribution.

We used CD to assess the overall folding of the target proteins and ensure that they maintain their structure during our experimental conditions. These experiments revealed that BceR and GraR share similar CD signature, indicating that any difference in their activities is not due to abnormal folding of the proteins in our experiments. GST-BceS and GST-GraS also share similar CD signature, indicating that they, too, share a similar folding pattern and any difference in their activities is not due to abnormal folding of the proteins in our experiments ( Figure 11).

### DNA-binding activity of GraR

**Figure 12.**
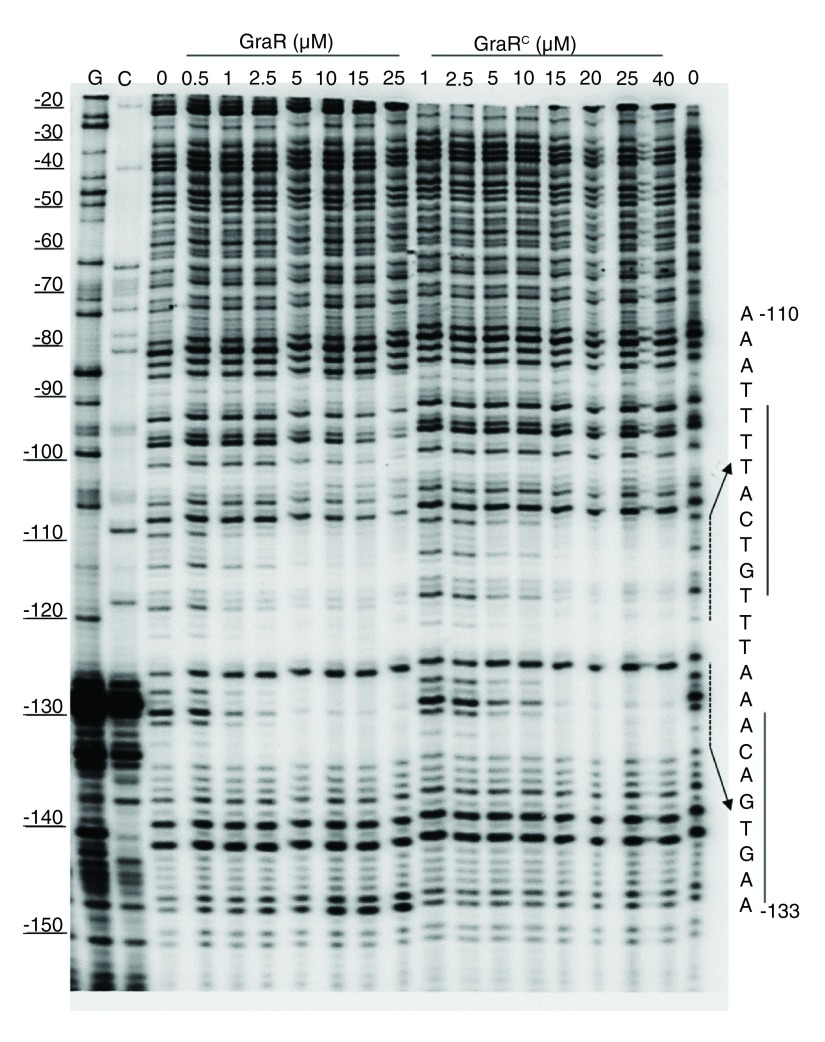
DNase I footprint analysis of *P _vraFG_* with GraR and GraR ^C^ (coding strand). *P _vraFG_* (10 ng) labeled on the coding strand was incubated with increasing concentrations of GraR or GraR ^C^ and subjected to DNase I. The DNA sequence protected by GraR is shown on the right. The dashed lines indicate the binding sites, and the solid lines show the palindromic sequence in DNA, as suggested by Dintner *et al.* ^21^.

**Figure 13.**
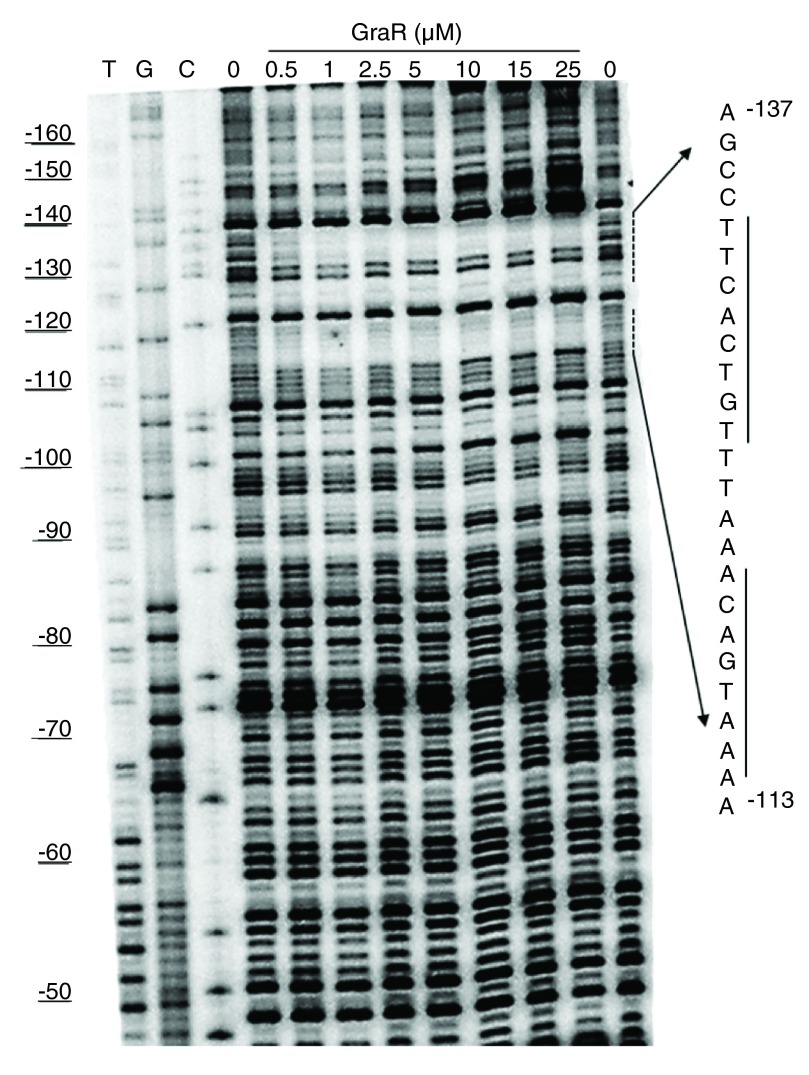
DNase I footprint analysis of *P _vraFG_* with GraR and GraR ^C^ (non-coding strand). *P _vraFG_* (10 ng) labeled on the non-coding strand was incubated with increasing concentrations of GraR or GraR ^C^ and subjected to DNase I. The DNA sequence protected by GraR is shown on the right. The dashed lines indicate the binding sites, and the solid lines show the palindromic sequence in DNA, as suggested by Dintner *et al.* ^21^.

To confirm that GraR is a functional protein in our study, we assessed the DNA-binding activity of this protein through DNase I footprinting experiments. These experiments showed that GraR bound to *P*_*vraFG*_ and protected a specific ~24 bp region located 110 bp upstream of the transcription starting point on the coding strand ( Figure 12). On the non-coding strand, the protected region was found 114 bp upstream of the *P _vraFG_* transcription starting point ( Figure 13). The protected region overlapped with the proposed GraR DNA-binding sequence ^15, 21^. We calculated the binding affinity of GraR for the target DNA (expressed as the dissociation constant *K*_d_) as the concentration of GraR that provided 50% protection from DNase I. Intensities of four bands, in the protected region, were measured at different GraR concentrations using ImageJ software. The estimated *K*_d_ was 0.8 ± 0.2 µM. Another region of *P _vraFG_*, spanning from -109 to -88, was slightly protected by GraR at concentrations greater than 15 µM.

**Figure 14.**
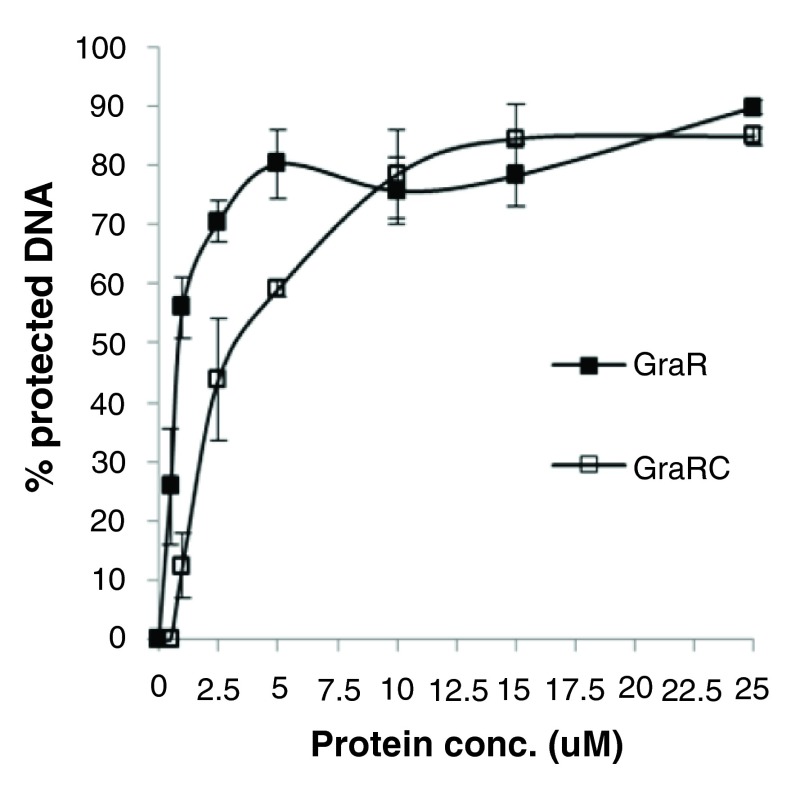
Quantification of DNAse I footprinting data ( Figure 12). Percentage of protected DNA in the region -110- to -133 is plotted against protein concentration used in the assay. We used the intensity of four most prominent bands in the GraR-protected region as measured using ImageJ (NIH software) and averaged them out to calculate the percentage of protected DNA. Error bars indicate standard error of mean calculated from three independent experiments.

GraR ^C^ protected similar regions of *P _vraFG_* as full length GraR ( Figure 2); however, it protected these regions at higher GraR ^C^ concentrations, indicating that the N-terminal domain has a role in the interaction of GraR with DNA probably mediating dimerization of GraR. The estimated *K _d_* value for GraR ^C^ was 2.4 ± 0.5 µM ( Figure 14). GraR did not protect any region on the *graRS* promoter (data not shown), which corroborates previous findings that GraR does not regulate expression of its operon ( *15*) and furthermore confirms that GraR is a functional protein in our experiments.

Data for signal transduction mechanism of GraSR in *Staphylococcus aureus* Files containing the CD data for GraS, GraR and its different constructs, GraX and BceR ( Figure 11) are provided. The averaged raw data for Figure 2b that were used to calculate the rate constant for phosphorylation are also provided.

## Discussion

The GraSR TCS, the VraFG ABC transporter and the accessory factor GraX are proposed to form a five-component system to control the response to CAMPs in *S. aureus* ^12^. The ABC transporter is essential to CAMP sensing but does not play a role in the resistance to CAMPs. VraG, a permease composed of 10 transmembrane domains, has a large extracellular domain that is proposed to directly sense CAMPs and transduce intracellularly the signal through GraSR ^12^. In addition, the accessory protein of the *graXRS* operon, GraX, is involved in both signaling of and resistance to CAMPs ^12^, but its function is not known. GraS is involved in signaling and GraR is involved in CAMPs resistance by directly regulating the genes that are responsible for D-alanylation of wall teichoic acid and lysinylation of phosphatidyl-glycerol within the cell membrane ^6, 9, 10^.

Since GraS and GraR constitute a TCS, it is supposed that upon intercepting a signal the GraS kinase will be activated through a trans-autophosphorylation process, whereby a conserved histidine residue of GraS will undergo phosphorylation. This signal will presumably be further transduced intracellularly through a second phosphorylation step, whereby the phosphoryl group of GraS will be transferred to GraR, resulting in its activation and initiation of the downstream regulatory steps. GraS lacks a bona-fide extracellular sensor domain, and this is considered as evidence that GraS is not directly involved in sensing CAMPs ^22, 23^, however, mutations at the extracellular loop that connects that two transmembrane helices of GraS have been reported to affect CAMP resistance ^8, 10^, suggesting otherwise.

The cytoplasmic domains of histidine kinases have been considered good models to study the autokinase/kinase activities of HKs ^18^. We show that the cytoplasmic domain of GraS does not have autophosphorylation activity. By contrast, a similar histidine kinase, BceS, undergoes autophosphorylation. Similar regions of GraS and BceS were cloned guided by their sequence homology ( Figure S1). Their CD spectra showed that both proteins share the same folding ( Figure 11). Hence, any difference between these two proteins in terms of their autophosphorylation activities should be considered an indication of their differences in function, only, and not misfolding of GraS. This begs the question, if GraS does not have autophosphorylation activity and it is not the CAMP sensor, how is the signal transduced through GraSR?

Our study shows that BceSR behaves as a typical TCS, i.e. BceS has autophosphorylation activity and it phosphorylates its cognate RR. Similarly to GraSR, BceSR also depends on an ABC transporter, BceAB, for sensing bacitracin, but unlike GraSR no auxiliary protein is required for signal transduction through BceSR ^14^. The dependence of CAMP signaling on the accessory protein GraX and our findings that GraX requires the DHp domain of GraS for interaction and it interacts with VraF, and in turn VraF requires the linker region of GraS for interaction, suggest that both these proteins may be involved in the activation of GraS kinase activity.

Our study shows that GraS interacts weakly with GraR, by contrast, BceS interacts strongly with BceR. The latter observation indicates that in a typical TCS, where HK phosphorylates its cognate RR, the unphosphorylated HK interacts with its cognate RR and that lack of interaction between GraR and GraS may be due to an improper alignment of structural elements in the DHp region known to determine the interaction between HK and its cognate RR ^24^. Notably, both GraR and BceR, and GraS and BceS share very similar CD signatures removing any doubt that any difference in function between these pairs is due to misfolding of GraR and GraS.

*In vivo* studies showed that deletion of *graS* prevents the *S. aureus* response to CAMPs ^12, 15^, which suggests that GraS plays a role in the regulation of GraR activity. This role of GraS is further confirmed by our finding that GraR does not undergo phosphorylation by acetyl phosphate, ruling out that this small-molecule phosphate donor could phosphorylate GraR *in vivo*. Notably, the interaction of GraX with GraS/R may result in increased local concentration of GraR, which in turn may forge interaction between GraS and GraR. Further, the dimeric state of GraX observed in our study may accommodate the observed interactions of GraX with GraS, GraR and VraF suggesting that GraX may serve as a scaffold, bringing several partners in close proximity.

The formation of a potential complex among GraS, GraX, GraR, and VraF may also serve to regulate the CAMPs signal transduction process. The linker that connects the two transmembrane-spanning α-helices of GraS is believed to reside within the lipid bilayer membrane of the cell due to its short length (nine amino acids) and as such, it may only be able to detect stimuli from within or at the membrane ^22^. However, because the conditions in and around the membrane constantly change due to cell growth and trafficking on both sides, it might be challenging for the GraS membrane-embedded linker to reliably sense the signal and initiate the signaling process. The association of GraS with the ABC transporter, which possesses a longer extracellular linker, offers the kinase an accurate reading of the signal among many look-alike signals. This hypothesis is supported by the report that GraSR-VraFG responds to selective CAMPs ^11^. Likewise, BraSR-BraDE and BceSR-BceAB respond to selective molecules. In contrast, VraSR and LiaSR TCSs, respectively in *S. aureus* and *B. subtilis*, which are not dependent on an ABC transporter for signaling, respond to cell wall damage caused by different classes of antibiotics that target cell wall biosynthesis ^14, 25^.

In conclusion, our study shows that the cytoplasmic domain of GraS does not have autophosphorylation activity, unlike that of BceS, and there is a weak interaction between GraS and its cognate response regulator GraR. These observations suggest that GraSR may not support a signal transduction process on its own. The tight interactions of GraX with GraS, GraR, and VraF suggest that GraX may serve as a scaffold, where VraF, GraS, and GraR dock to increase the local concentration of proteins and forge further interactions among them. This complex formation may enable phosphorylation of GraS. Upon activation of GraS, the extracellular signal may be transduced to GraR through a phosphotransfer process, leading to the initiation of downstream events.

## Data availability

F1000Research: Dataset 1. Data for signal transduction mechanism of GraSR in *Staphylococcus aureus*, 10.5256/f1000research.5512.d37228 ^26^
