# Supplementary material for: Diversity of two-component systems: insights into the signal transduction mechanism by the  Staphylococcus aureus two-component system GraSR
Source: F1000Res. 2014 Nov 17;3:252. Originally published 2014 Oct 27. [Version 2] doi: 10.12688/f1000research.5512.2 (PMC4314665; doi:10.12688/f1000research.5512.2)
Supplement: Peer review history

# Peer review: Referee response for version 2

**DOI:** 10.5256/f1000research.6135.r7482

**Published:** 2015-02-02

**Authors:** Michael Otto

**Affiliations:** 1 National Institute of Allergy and Infectious Diseases, National Institutes of Health, Bethesda, MD, USA

The GraRSX (ApsRSX) system is a CAMP-sensing system that recently has been shown to also require the presence of VraFG, whose expression it regulates, for signal transduction. Muzamal *et al.* here performed several experiments to further delineate details of the signal transduction process of this system.

They analyzed interactions between the different proteins (notably, however, lacking investigation of VraG), which led them to conclude that GraX may serve as a “scaffold” between the two systems. They found also that GraS, the histidine kinase part of the GraRS TCS, lacks autophosphorylation activity.

I have several major problems with this report. The conclusions from new findings are not sufficiently backed by conclusive evidence, often based on negative results; and there are many open ends in the report. The overall picture of the mechanism of GraRSX/VraFG signaling is biased. Finally, the responses to major and valid concerns of the previous reviewer one are unsatisfactory.

1. The finding that GraX is required for signal transduction and may serve as a “scaffold” between the Gra and Vra proteins is not novel. The authors fail to cite, like on many other occasions, evidence derived from the first investigation of the ApsRSX (GraRSX) system (ref. 10), which was undertaken in detail in *S. epidermidis*, whose system shares high similarity with that of S. aureus. It was shown in that study that ApsX (GraX) is needed for CAMP sensing/signal transduction. Furthermore, Falon *et al*. (ref. 12) already performed experiments clearly indicating that GraX has that scaffold function. This main message of the report thus lacks novelty. Admittedly, the authors for the first time used protein-based methods to substantiate that model – however, the used single method appears insufficient and technically problematic – see mainly point 2.
2. Main conclusions are based on protein-protein interaction studies by pull-down assays. As correctly criticized by reviewer 2, who asked for further experiments with different methods analyzing interaction, results from such assays are difficult to interpret, as they are heavily dependent on the used conditions – especially when basing conclusions on negative results. This is the heart of the study, and to substantiate these results and provide a significant scientific advance, other methods must be used - especially given that Falon *et al. *already investigated those protein interactions with genetic tools and came to similar conclusions.
3. The authors fail to correctly and sufficiently cite relevant findings from the literature, leading them to a biased presentation of the CAMP recognition mechanism. They state that GraS is not involved in CAMP sensing, despite clear evidence indicating otherwise. They only cite ref. 8 in that regard, in which it was shown that changing the extracellular loop of GraS results in changed CAMP sensing. They do, again, not cite ref. 10, in which experiments were performed further substantiating that role of GraS. There is certainly controversy regarding this question, but the way it is presented in this manuscript is biased, owing to the selective citing of previous studies.
4. The authors draw conclusions about the lack of autophosphorylation activity of GraS from the investigation of a truncated GraS and from comparison to an only distantly (35% similarity) related system. This is not conclusive. Autophophorylation may need the whole protein and only because BceS undergoes autophophorylation under the tested conditions does not mean GraS would not under different ones.
5. As pointed out already, important findings from the first study in *S. epidermidis* are not included and appropriately discussed. Furthermore, there has been evidence (ref. 8) that vraFG has an impact, although minor, on CAMP resistance – in contrast to what the authors state. Here again, different findings in the literature are reported in a selective fashion.
6. The previous reviewer one had several important remarks, most of which were not addressed in a satisfactory fashion. That reviewer noted that conclusions from the comparison to the Bce system are difficult to interpret, owing to a lack of similarity and a difference in function. I concur with that notion – only because a remotely similar system shows an effect, and Gra proteins do not,  does not mean that Gra proteins would not show an effect under different conditions. The authors draw conclusions from negative results. Second, the reviewer asked for other types of interaction studies to substantiate the results from the pull-down experiments (see above). This was not addressed. Third, the authors did not address VraG, simply stating it is not amenable to their type of studies as it is a membrane protein. Then they should perform other studies, as requested. Fourth, the reviewer asked for footprinting assays to be performed with phosphorylated protein. I completely concur that it is vital, especially given the model the authors propose, to perform these studies with clearly phosphorylated or de-phosphorylated protein. The authors simply state in their response that GraR does not undergo auto-phosphorylation by acetyl phosphate. If the authors cannot achieve phosphorylation by acetylphosphate, they should at least completely de-phosphorylate and show that binding is affected by phosphorylation. Fifth, the reviewer had a valid concern stating that the data in Fig. 9 should be presented like those in Fig. 10 to clearly demonstrate the absence of non-specific binding. Here again, the authors simply stated that they believe the data in Fig. 10 are enough to rule out such non-specific binding. However, there are completely different protein-protein interactions that are investigated in Fig. 9.

Further remarks:

The authors should at least mention the other name of the system – ApsRSX. It was in the first report that demonstrated CAMP sensing of the system in *S. epidermidis *that it was named that way.

In my opinion, the authors mainly must provide evidence for presence/lack of protein-protein interactions by a second method, in addition to answering appropriately to the other technical concerns and presenting a non-biased discussion about the sensing system.

I have read this submission. I believe that I have an appropriate level of expertise to confirm that it is of an acceptable scientific standard, however I have significant reservations, as outlined above.

# Peer review: Referee response for version 2

**DOI:** 10.5256/f1000research.6135.r6730

**Published:** 2014-11-26

**Authors:** Jonathan D. Partridge

**Affiliations:** 1 Section of Molecular Genetics and Microbiology and Institute of Cellular and Molecular Biology, University of Texas at Austin, Austin, TX, USA

Upon examination of the revised manuscript I am satisfied that my concerns were addressed and queries answered. Revisions made by the authors make the manuscript suitable for approval.

I have read this submission. I believe that I have an appropriate level of expertise to confirm that it is of an acceptable scientific standard.

# Peer review: Referee response for version 1

**DOI:** 10.5256/f1000research.5884.r6605

**Published:** 2014-11-10

**Authors:** Jonathan D. Partridge

**Affiliations:** 1 Section of Molecular Genetics and Microbiology and Institute of Cellular and Molecular Biology, University of Texas at Austin, Austin, TX, USA

The work by Muzamal and co-workers is an in depth study of the *Staphylococcus *two-component system GraRS, its auxiliary protein GraX and associated ABC transporter system vraG. The work is thorough and shows a methodical approach to characterizing the players in this system, however a few issues need to be addressed.

**Major comments:**

1. Considering how detailed the work is, it is surprising that VraG, co-transcribed with VraF and under the regulatory control of GraR, is not investigated. Why?
2. If components of this system sense and bind CAMPs (page 3) to effect a change, perhaps some of these binding studies should have been done in the presence as well as absence of CAMPs.

 

**Minor comments:**

 

1. pg. 4, Protein concentrations determined by Bradford assay, a reference should be included.
2. pg. 5, "GraX fractions were pooled and concentrated". How were proteins concentrated?
3. pg. 5, cell cultures were cooled to 4°C before IPTG induction at 16°C for 16 h. Why?
4. pg. 6, DNase footprinting experiments, +28 to -168 was used, what was the justification? Has the promoter architecture been mapped to solely this region? If so, a reference should be added.
5. pg. 7, "cytoplasmic domains of histidine kinases have been used as model to study...", should be “model **s**” **.**
6. Why does the ratio of protein:protein used in experiments vary?
7. The quality of some of the gel figures are perfectly clear whilst some are poor, exacerbated when the document is printed.  More consistency in quality would improve interpretation of the manuscript.
8. Due to the large number of expression vectors used in the study, a small table should be added to note the designation, host, resistance, induction etc, of those plasmids used and those plasmids made for reader clarity and reference.
9. Some of the figures referred to in the manuscript do not match to the correct figures, this should be thoroughly checked.

I have read this submission. I believe that I have an appropriate level of expertise to confirm that it is of an acceptable scientific standard, however I have significant reservations, as outlined above.

# Response

**Published:** 2014-11-10

**Authors:** Dasantila Golemi-Kotra

**Affiliations:** York University, Toronto, Canada

Thank you very much for the critical reading of our work. We have addressed your comments below.

**Major comments:**

1. *"Considering how detailed the work is, it is surprising that VraG, co-transcribed with VraF and under the regulatory control of GraR, is not investigated. Why?"*
  VraG is a membrane embedded protein with 10 membrane spanning a-helices. Hence this protein is not amenable to our experiments.
2. *"If components of this system sense and bind CAMPs (page 3) to effect a change, perhaps some of these binding studies should have been done in the presence as well as absence of CAMPs."*
  This is a good point. Our GraS construct does not carry the membrane bound domain and the other proteins are cytoplasmic proteins. The ABC transporter VraFG is shown not to be a transporter for the CAMPS. Nonetheless, we included indolicidin in the phosphorylation experiments for GraS and GraR as well as DNA-binding experiments with GraR. We did not see any affect. These data are not shown and not discussed as they do not provide any important insight to this study.

**Minor comments:**

1. *"pg. 4, Protein concentrations determined by Bradford assay, a reference should be included."*
  We used the Bradford reagent obtained from GE Healthcare, and used the manufacturer’s instructions for its use. The text is corrected to reflect this fact.
2. *"pg. 5, "GraX fractions were pooled and concentrated". How were proteins concentrated?"*
  This information is added to the main text. We use an Amicon Stirred Cell.
3. *"pg. 5, cell cultures were cooled to 4°C before IPTG induction at 16°C for 16 h. Why?"*
  Under this conditions, we obtained an optimum production of the soluble protein. This could be due to the decrease of gene expression rate at the low temperature, which may result in a smaller chance of newly synthesized protein to be trapped as inclusion bodies.
4. *"pg. 6, DNase footprinting experiments, +28 to -168 was used, what was the justification? Has the promoter architecture been mapped to solely this region? If so, a reference should be added."*
  We used the entire intergenic region between the *graXSR* operon and the upstream operon in *S. aureus* Mu 50 genome, and part of the coding region of *graXSR* operon. Earlier studies on the proposed GraR binding sequence are provided in the text, ref. 15 and 21 (page 6 and 13).
5. *"pg. 7, "cytoplasmic domains of histidine kinases have been used as model to study...", should be “model **s**”."*
  This is corrected as indicated.
6. *"Why does the ratio of protein:protein used in experiments vary?"*
  Stock solutions for each protein were different.
7. *"The quality of some of the gel figures are perfectly clear whilst some are poor, exacerbated when the document is printed.  More consistency in quality would improve interpretation of the manuscript."*
  We regret the apparent inconsistency in figure quality. This is due to the protective measurements that we need to take when taking pictures of radioactive SDS-PAGE that are stained with Coomassie (in order to see the protein bands); we have to use a plastic wrap.
8. *"Due to the large number of expression vectors used in the study, a small table should be added to note the designation, host, resistance, induction etc, of those plasmids used and those plasmids made for reader clarity and reference."*
  We have used only three expression vectors: pET26, pGEX-4T and pT7-MAT-tag-Flag1.
9. *"Some of the figures referred to in the manuscript do not match to the correct figures, this should be thoroughly checked."*
  We have made the corrections wherever necessary.

# Peer review: Referee response for version 1

**DOI:** 10.5256/f1000research.5884.r6514

**Published:** 2014-10-31

**Authors:** Ambrose Cheung

**Affiliations:** 1 Department of Microbiology and Immunology, Dartmouth College, Hanover, NH, 03755, USA

This is a biochemical study that dissects the interaction of a two component regulatory system (GraRS) with a third component (GraX) and its downstream ABC transporter system composed of a permease (vraG) and an ATPase (VraF). The major contribution of this paper is to ascertain the interaction of GraX with VraF and GraS. In addition, VraF also interacts with GraS, thus implying a complex system of interaction likely involving at least four proteins. However, the enthusiasm for this manuscript is tempered by several major deficiencies that need to be amended.

Major issues:

1. The comparison of BceRS to GraRS is invalid because the correct comparison should be between BraRS (SA2417-2418) and BceRS. Similar to BceRS, BraRS is a TCRS that, together with BraDE (efflux pump system), is required for bacitracin resistance in *S. aureus *(ref. 23).
2. The interaction of different protein depends on pulldown study where it appears that there is likely to be protein overload to the system (e.g. GraR appears in large amount of the unbound fraction).  This reviewer believes the amount of protein used as binding partner should be reduced (to avoid non-specific interaction) and then detected by immunoblots.
3. The authors totally ignored the contribution of VraG
4. The critical protein binding studies in Fig. 9 should be displayed as in Fig. 10 where data on unbound, washed and eluted fractions should be presented.  Also, control protein should be included in these data ensure there is no non-specific protein interaction, especially when proteins are used at a relatively high quantities.
5. It would be useful to confirm different protein binding studies (Fig. 9) with another method such as Biacore, FTIR or two-hybrid system.
6. Cull back on negative data and mention in the text.
7. The authors should consider using phosphorylated GraR on the DNAse I footprinting assay.

 

Minor points:

1. ABC transporter senses CAMP (page 3) – this is more controversial. I am not sure how the transporter senses since this is not a typical sensing system, especially when this is an efflux system with no ligand binding protein exposed to the outside.
2. What is the control for Figure 3 in this system?
3. In Fig. 7C, what is the elution profile of the “unphosphorylated VraR”?
4. There appears to be mislabeling in Fig. 9G (GraX instead of GraS). Similarly, Fig. 10A is also mislabeled (GraR instead of GraX).
5. Explain the rationale for the different protein ratios in the protein binding experiments.
6. On p.13, third paragraph, GraRc protected similar….. (Fig. 6).  The figure is wrongly references.
7. In the discussion, the author mentions that “GraS lacks a bona-fide extracellular sensor domain”. There is a short extracellular loop in GraS. How long does it have to be to be “bona fide”?
8. Could binding of GraS by CAMP activate phosphorylation?

I have read this submission. I believe that I have an appropriate level of expertise to confirm that it is of an acceptable scientific standard, however I have significant reservations, as outlined above.

# Response

**Published:** 2014-11-10

**Authors:** Dasantila Golemi-Kotra

**Affiliations:** York University, Toronto, Canada

Thank you very much for your careful review of our paper. I have addressed your concerns in the order they were raised.

**Major issues:**

1. *"The comparison of BceRS to GraRS is invalid because the correct comparison should be between BraRS (SA2417-2418) and BceRS. Similar to BceRS, BraRS is a TCRS that, together with BraDE (efflux pump system), is required for bacitracin resistance in S. aureus (ref. 23)."*
  It is important to note that GraSR proteins share the highest sequence identities with BceSR proteins, rather BraSR proteins, and that is why for the purpose of characterization of GraSR activities we refereed to this two-component system, rather than BraSR.
2. *"The interaction of different protein depends on pulldown study where it appears that there is likely to be protein overload to the system (e.g. GraR appears in large amount of the unbound fraction).  This reviewer believes the amount of protein used as binding partner should be reduced (to avoid non-specific interaction) and then detected by immunoblots."*
  This is a valid concern. I would like to make the point, as to clarify our experimental conditions and remove any misunderstanding, the protein concentration for the “bait” does not exceed 10-15 µM and the protein concentration for the “prey” is kept between 10-30 µM (1:1 or 1:2 ratio), with the exception when we did not see interaction under these conditions and we had to increase concentration of the “prey” protein to 5-fold more than the “bait” protein (the case of GraS:GraR). These protein concentrations are actually at the low end of any other technique used for binding experiments. In addition, as our data show, under our experimental conditions GraR does not bind to GraS at 2:1 ratio and GraX and VraF do not interact with all the GraS constructs. As well, the control experiments demonstrate that under our working conditions there are no non-specific interactions.
3. *"The authors totally ignored the contribution of VraG"*
  VraG is an important player in the signal transduction process of GraSR. It is a membrane embedded protein that is not amenable to the methods used in our study.
4. *"The critical protein binding studies in Fig. 9 should be displayed as in Fig. 10 where data on unbound, washed and eluted fractions should be presented.  Also, control protein should be included in these data ensure there is no non-specific protein interaction, especially when proteins are used at a relatively high quantities."*
  We believe that Figure 10 addresses the issues of non-specificity that may arise from these experiments and addition of other figures will be redundant.
5. *"It would be useful to confirm different protein binding studies (Fig. 9) with another method such as Biacore, FTIR or two-hybrid system."*
  We have tried isothermal titration calorimetry experiments. But this method, like FTIR and Biacore, suffers from the need of having to use higher protein concentrations in the assay which leads to aggregation of our target proteins.
6. *"Cull back on negative data and mention in the text."*
  It has been the Journal’s advice to include the data wherever possible and we followed their advice.
7. *"The authors should consider using phosphorylated GraR on the DNAse I footprinting assay."*
  We show that GraR does not undergo phosphorylation by acetyl phosphate, Figure 8.

**Minor points:**

1. *"ABC transporter senses CAMP (page 3) – this is more controversial. I am not sure how the transporter senses since this is not a typical sensing system, especially when this is an efflux system with no ligand binding protein exposed to the outside."*
  The participation of the ABC transporters in signaling is a new model that has been proposed recently (Ref. 21 and 22). Hence, we have presented in our article both views that are currently present in the literature, that of GraS serving as the sensor (Ref. 8,11) and that of the extracellular region of VraG serving as the sensor (Ref. 12).
2. *"What is the control for Figure 3 in this system?"*
  Figure 2 is the control for this experiment as both proteins were subjected under the same phosphorylation conditions.
3. *"In Fig. 7C, what is the elution profile of the “unphosphorylated VraR”?"*
  We have published these data in Ref 18 (Supplemental data).
4. *"There appears to be mislabeling in Fig. 9G (GraX instead of GraS). Similarly, Fig. 10A is also mislabeled (GraR instead of GraX)."*
  Thank you for noting them. We have accordingly done the corrections.
5. *"Explain the rationale for the different protein ratios in the protein binding experiments." *
  The slight deviations on the protein ratios from 1:1 ratio in the pull-down experiments was due the actual stock protein concentrations that were available after protein purification/concentration. Some of these proteins are prone to aggregation at relatively low concentrations e.g. 30-50 µM, such as GraS and GraX, and/or not produced at large quantities such as VraF.
6. *"On p.13, third paragraph, GraRc protected similar….. (Fig. 6).  The figure is wrongly references."*
  Corrected accordingly.
7. *"In the discussion, the author mentions that “GraS lacks a bona-fide extracellular sensor domain”. There is a short extracellular loop in GraS. How long does it have to be to be “bona fide”?"*
  This is a good question. There is a discussion on this matter in literature (Ref. 22). There is still room to further investigate this matter.
8. *"Could binding of GraS by CAMP activate phosphorylation?"*
  There are studies that indicate that GraS is directly involved in sensing of CAMPs (Ref. 8, 11), which cannot be ignored. Our work is ongoing to further explore the signaling mechanism by GraS.
